# The impact of leisure activities on older adults’ cognitive function, physical function, and mental health

**DOI:** 10.1371/journal.pone.0225006

**Published:** 2019-11-08

**Authors:** Giovanni Sala, Daniela Jopp, Fernand Gobet, Madoka Ogawa, Yoshiko Ishioka, Yukie Masui, Hiroki Inagaki, Takeshi Nakagawa, Saori Yasumoto, Tatsuro Ishizaki, Yasumichi Arai, Kazunori Ikebe, Kei Kamide, Yasuyuki Gondo

**Affiliations:** 1 Institute for Comprehensive Medical Science, Fujita Health University, Toyoake, Japan; 2 Institute of Psychology, University of Lausanne, Lausanne, Switzerland; 3 Centre for Philosophy of Natural and Social Science, London School of Economics and Political Science, London, United Kingdom; 4 Tokyo Metropolitan Institute of Gerontology, Tokyo, Japan; 5 Graduate School of Science and Technology, Keio University, Yokohama, Japan; 6 National Center for Geriatrics and Gerontology, Aichi, Japan; 7 Graduate School of Human Sciences, University of Osaka, Osaka, Japan; 8 Center for Supercentenarian Medical Research, Keio University, Tokyo, Japan; 9 Graduate School of Dentistry, Osaka University, Osaka, Japan; 10 Graduate School of Medicine, Osaka University, Osaka, Japan; Nathan S Kline Institute, UNITED STATES

## Abstract

Engagement in leisure activities has been claimed to be highly beneficial in the elderly. Practicing such activities is supposed to help older adults to preserve cognitive function, physical function, and mental health, and thus to contribute to successful aging. We used structural equation modeling (SEM) to analyze the impact of leisure activities on these constructs in a large sample of Japanese older adults (*N* = 809; age range 72–74). The model exhibited an excellent fit (CFI = 1); engaging in leisure activities was positively associated with all the three successful aging indicators. These findings corroborate previous research carried out in Western countries and extend its validity to the population of Eastern older adults. Albeit correlational in nature, these results suggest that active engagement in leisure activities can help older adults to maintain cognitive, physical, and mental health. Future research will clarify whether there is a causal relationship between engagement in leisure activities and successful aging.

## Introduction

Leisure activities (hereafter LA) can be defined as activities people engage in during free time [1]. Engagement in LAs has been found to be positively associated with cognitive function, physical function, and mental health in late adulthood and in the elderly. The possible protective effects of LA engagement against aging-related decline have thus been the object of investigation in the last two decades.

Of these three outcomes, preserved cognitive function has received most attention and the link with LA engagement in the elderly is well established [2–5]. Three possible explanations have been proposed for the observed relationship between cognitive function and LA engagement. First, practicing mentally challenging activities (e.g., music, board games, video games, and brain training) may enhance overall cognitive function [6]. However, this idea has received little adequate empirical support [7–9] in the general population. Second, people exhibiting superior overall cognitive function may be more likely to engage in LAs that are cognitive demanding. This hypothesis has been corroborated by numerous studies in the field of chess and music [10,11]. Finally, engaging in intellectually demanding LAs may slow down cognitive decline. This idea relies on the so-called “use it or lose it” hypothesis according to which engaging in intellectually demanding activities helps to preserve cognitive function in the elderly [12,13]. This hypothesis has received some support by studies implementing dual-change approaches to test for causality between LA engagement and preserved cognitive function [14,15].

Compared to the link between LA engagement and cognitive function, the impact of LA engagement on physical function has been less studied [16]. Most research has focused on the adverse effects of illness and injuries on LA engagement. Reduced LA engagement has, in turn, detrimental effects on indicators of mental health such as well-being and life satisfaction [17]. The field has thus paid more attention to how physical function influences LA engagement rather than vice-versa. Another line of research has examined the effects of physical activity and LAs (as independent variables) on cognitive function [12,18].

Finally, LA engagement seems to be related to mental health as well. Mental health aspects such as well-being and life satisfaction have been found to positively correlate with LA engagement in several studies (for a review, see [17]). Studies implementing a longitudinal design have confirmed these findings [19]. Nonetheless, the amount of robust experimental evidence is still modest [20].

Considering its positive effects for key dimensions of functioning in older age, LA engagement seems to have an important role for *successful aging*. In their seminal article, Rowe and Kahn [21] introduced the concept of successful aging in opposition to *usual aging*. While usual aging emphasizes the non-pathological aging (e.g., absence of disease), successful aging captures an optimal aging process. Successful aging is, according to Rowe and Kahn, best characterized by the concurrent presence of three dimensions, namely high cognitive and physical function, low probability of disease and disability, and active engagement in life. The latter captures the involvement in productive and social activities, giving those activities a similar importance as health and functioning for successful aging. The importance of activities for successful aging has also been stressed very early on in other seminal theories, including activity theory by Havighurst, Neugarten, and Tobin [22]. Such more multidimensional conceptualizations of successful aging have resulted in complementing prior approaches by including mental-health related dimensions such as well-being [17,23–25]. Here, we investigate the relationship between engagement in LAs and fundamental dimensions of successful aging such as cognitive function, physical function, and mental health.

### The present investigation

This study examines the impact of engagement in LAs on cognitive function, physical function, and mental health in a group (*N* = 809) of Japanese older adults. As most of the studies in the field are based on samples from Western countries (e.g., US and Germany), little is known about this issue in non-Western countries. The present study aims to fill this gap by adding new information from a country of the Far East (i.e., Japan). Moreover, prior studies usually suffer from limitations such as relying on subjective measures of cognitive and physical function [26,27] and dichotomization of continuous variables [27,28]. Also, the use of multiple-indicator latent variables, which is necessary to reduce measurement error and, hence, produce more reliable estimates, has been sporadic [2]. The present study employs objective measures of functioning as well as multiple indicators allowing complex modeling and controls for measurement error.

Furthermore, previous studies of both Western and Eastern populations have focused on the association between LA engagement and other components of successful aging (e.g., cognitive function) one at a time. By contrast, here we study the relationship between LA engagement and cognitive function, physical function, and mental health in a single model. This approach allows us to examine not only the direct effects of LA engagement on indicators of successful aging, but also how cognitive function, physical function, and mental health influences each other. Recent research into the field has in fact shown that mutual relationships occur between cognitive function, physical function, and mental health [17,29–31]. However, these bi-directional effects have never been studied in relationship with LA engagement.

We aim to address the above limitations with Structural Equation Modeling (SEM). An SEM model allows us to analyze the impact of LA engagement on all the variables of interest–cognitive function, physical function, and mental health–simultaneously (i.e., in a single SEM model). Such a model rules out potential confounds (e.g., Type I error due to a missing covariate) and takes into account the bidirectional paths between the endogenous latent variables. This way, the model estimates the unique (i.e., not confounded by other correlated variables) impact of LA engagement on the other dimensions of successful aging. To the best of our knowledge, no such study has been carried out in this field so far. Also, SEM models can estimate latent constructs of interest (i.e., cognitive function, physical function, and mental health), which are psychometrically more precise and reliable than observed indicators (i.e., test and questionnaires scores). Finally, while the studies in the field have usually assessed physical function with self-report questionnaires [19], the indicators we use are objective measures.

To sum up, the study aims (a) to test the previous claims about the relationship between LA engagement and successful aging by employing a more robust and comprehensive modeling approach; (b) to extend the current empirical evidence–which is mostly on cognitive function–by examining the impact of LA engagement on less studied dimensions of successful aging such as physical function and mental health; (c) to quantify both direct and indirect effects of LA engagement; and (d) to extend the relatively small amount of data concerning the role played by LA engagement in the successful aging dimensions of Eastern populations.

## Materials and methods

### Participants

The study included a total of 809 Japanese participants (381 men and 428 women). The age range was 72 to 74. The data were retrieved from the second wave of the Septuagenarians, Octogenarians, Nonagenarians Investigation with Centenarians (SONIC) survey. These particular study cohort and wave were selected because they reported the most extensive and detailed LA-engagement questionnaire.

The SONIC is an ongoing survey whose main purpose is to identify the correlates of healthy aging. It includes both urban and rural (ratio 2:1) community-dwelling older adults. The participants were recruited from residential registries and contacted by postal mail. They gave their informed consent on site prior to starting the survey. For all the details about the SONIC survey, see [32].

### Variables

#### Leisure-activity (LA) engagement

This questionnaire included 158 yes/no items regarding the participant’s engagement in as many activities. The questionnaire was based on the questionnaires presented in Karp et al. and Jopp and Hertzog’s studies [4,33], and it was extended by adding common activities among Japanese older adults (e.g., playing shogi, practicing tai chi, and going to a public bath [onsen]). The questionnaire showed good internal consistency (Cronbach’s *α* = .88). The list of the activities is reported in the Supplemental materials available online. A latent factor representing LA engagement was extracted from the questionnaire with the ltm R package and used in the analyses [34].

#### Cognitive function

Three measures of cognitive ability were used to estimate a latent variable representing cognitive function: the Japanese version of the Montreal Cognitive Assessment (MoCA-J; [35]); the number series completion task from the Brief Test of Adult Cognition by Telephone as a measure for reasoning skills [36]; and the recall subtest of the Alzheimer's Disease Assessment Scale (ADAS; [37]). For all tests, a specific score was obtained, with higher values indicating higher capacity.

#### Physical function

Two measures of physical function from the Short Physical Performance Battery (SPPB; [38]) were administered: the participants’ gait speed and chair stand test. For both tests, performance indicators were captured in seconds, with fewer seconds to complete the task indicating better health functioning. The third measure was the 10-second open-close stepping test [39], for which a smaller number of steps to complete the task indicated better functional health.

#### Mental health

Three measures of mental health were administered: the Japanese versions of the WHO-5 well-being index questionnaire [40], positive section of the Positive and Negative Affect Scales (PANAS; [41]), and Satisfaction With Life Scale [42]. In all the indicators, higher numbers indicate better mental health.

#### Covariates

We included gender (male, female), education and wealth as covariates to control for their potential effect on LA engagement or its link to the successful aging outcomes. Education consisted of three levels indicating the highest degree achieved by the participant (1 = primary/middle school, 2 = high school, and 3 = university/college education). Wealth described the participant’s economic situation and included five levels (from 1 = difficult economic situation to 5 = very good economic situation). These two variables were added to assure that the effect of LA engagement was not confounded with SES-related variables such as education and wealth status. Finally, gender was used as a grouping variable to test for measurement invariance across males and females. The rationale of this choice is that SES variables (such as education and wealth) may not always be equally good predictors of the constructs of interests between males and females. For example, it is reasonable to suppose that, in the fifties, intellectually gifted males were more likely to advance in their studies than equally intellectually gifted females.

### Data preparation and analysis

#### Variables transformation and outlier treatment

All the continuous variables were normalized and standardized. Normalization reduces the inflation of absolute measures of fit (e.g., χ^2^) and incidence of outliers in the dataset. Also, normalization tends to linearize the relationship among multiple measures of the same construct [43], and thus reduces biases due to potential non-linear relationships between indicators. The normalization was run with the bestNormalize R package [44].

The normalized variables were inspected for possible outliers. A value was considered as an outlier if it fell outside of the following range [Q1–2.2*IQR; Q3 + 2.2*IQR], where Q1, Q3, and IQR were the first quartile, the third quartile, and the interquartile range of the variable, respectively [45]. Only two outliers were detected (both in the MoCA-J scores) and winsorized.

#### Power calculation

The statistical power for not-close fit hypothesis testing was estimated [43]. Assuming the null RMSEA = .050, alternative RMSEA = .010, and α = .050, the statistical power was very high (> 99%). The number of participants recruited was thus more than adequate for global fit testing and rejection of false models. These analyses were run with the semTools R package [46].

### SEM modeling

The lavaan R package [47] was used to run all the SEM models. An equality constraint between two factors (physical function and mental health) was added to identify the models. We built one model including all the variables. LA engagement, subjective wealth, and education were predictors of the three latent variables cognition, physical function, and mental health in the regression equations. Also, since some studies have provided evidence of a bidirectional relationship between physical function, cognitive function, and mental health [17,29,30], feedback loops were included between the latent variables. Due to the inclusion of two ordinal variables (education and subjective wealth), we chose the unweighted weighted least squares estimator (ULS). (An additional set of analyses was carried out with an alternative estimator [WLSM] that does not assume multivariate normality.) Gender was used for multiple-group analysis.

## Results

The descriptive statistics (untransformed means and standard deviations) are reported in Table 1.

**Table 1:** The descriptive statistics of the variables.

	Males (*N* = 381)		Females (*N* = 428)				Total (*N* = 809)		
*Variable*	*Mean*	*SD*	*Mean*	*SD*	*t-value*	*p-value*	*Mean*	*SD*	*Range*
LA Engagement	21.64	10.45	22.47	10.49	-1.12	.263	22.08	10.48	0–65
MoCA-J	23.41	2.92	23.95	2.96	-2.58	.010	23.69	2.95	12–30
ADAS Recall	14.91	3.71	16.61	3.74	-6.49	.000	15.81	3.82	5–26
Number Series	0.11	1.08	-0.21	1.05	4.29	.000	-0.06	1.07	-2.59–2.00
Chair Stand Test (s)	10.88	3.37	10.14	2.96	3.36	.001	10.49	3.18	3.78–32.57
Gait Speed (s)	2.47	0.57	2.41	0.51	1.63	.104	2.44	0.54	1.36–5.69
Stepping Test	25.19	6.42	26.40	5.75	-2.82	.005	25.82	6.10	6–46
WHO5	15.63	5.09	16.17	4.85	-1.54	.123	15.91	4.97	0–25
Positive Affect	10.38	2.76	11.29	2.57	-4.83	.000	10.86	2.70	3–15
Life Satisfaction	23.24	5.72	23.03	5.38	0.52	.602	23.13	5.54	5–35
Education	2.17	0.73	1.98	0.67	3.78	.000	2.07	0.71	1–3
Wealth	2.94	0.81	2.91	0.79	0.55	.584	2.92	0.80	1–5

**Fig 1:**
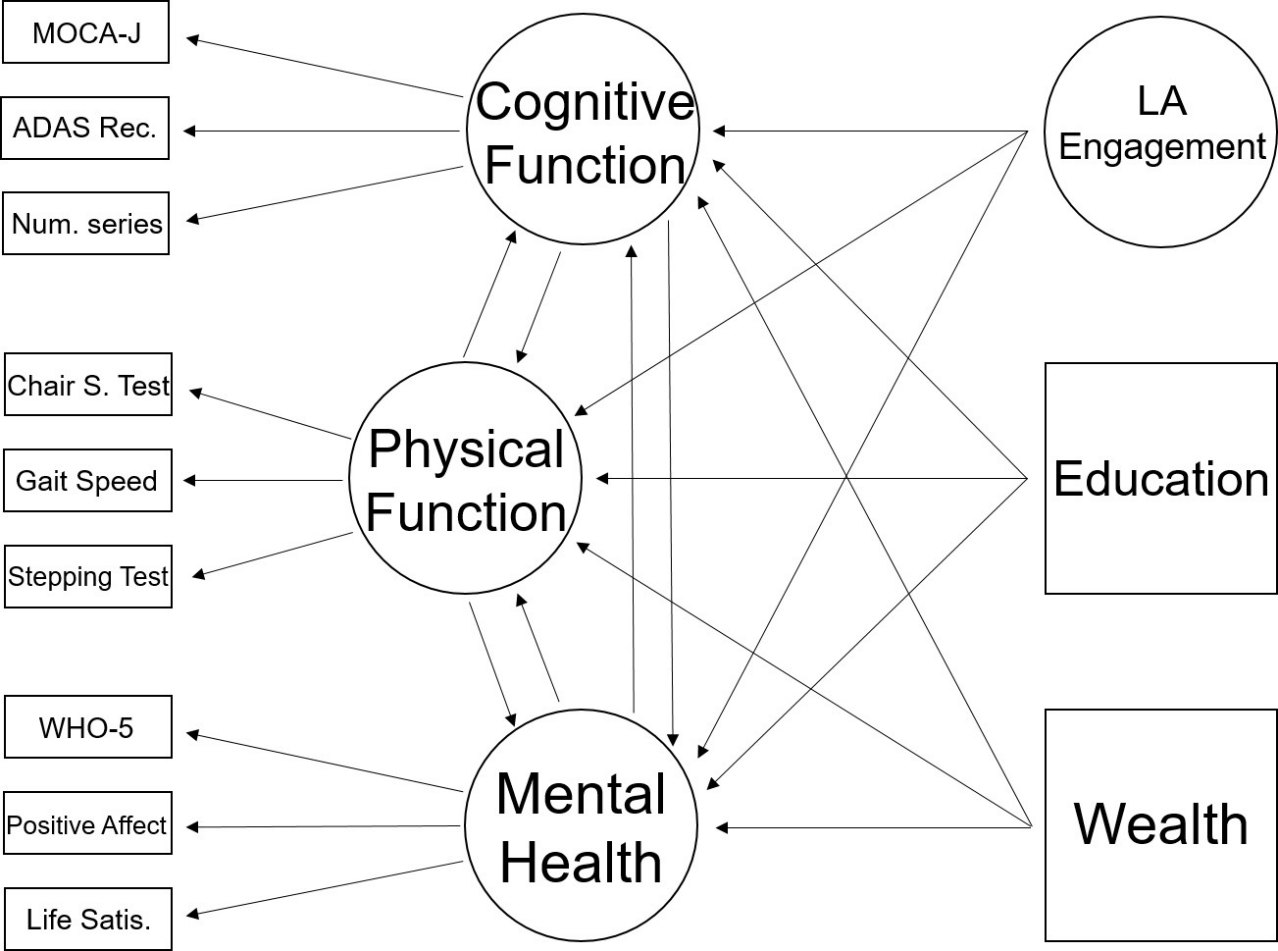
The SEM model. The squares and rectangles represent observed variables. The rectangles represent indicators for the latent variables (circles). The arrows represent the paths.

The SEM model in which LA engagement predicted cognitive function, physical function, and mental health as concurrent successful aging outcomes, and controlling for SES (i.e., education and wealth), proved to have an excellent fit: χ^2^(79) = 70.34, RMSEA = 0.000, SMRM = 0.037, CFI = 1.000. Comparative fit testing showed that this model outperformed the homologous model without the grouping variable (i.e., gender; CFI = 1.000). Measurement invariance analysis showed that the weak (metric) invariance was not rejected (*p* = .356). The strong (i.e., scalar) invariance hypothesis was rejected (*p* < .001), suggesting the presence of a differential additive response style [43] between genders. The model structure is in Fig 1.

**Fig 2:**
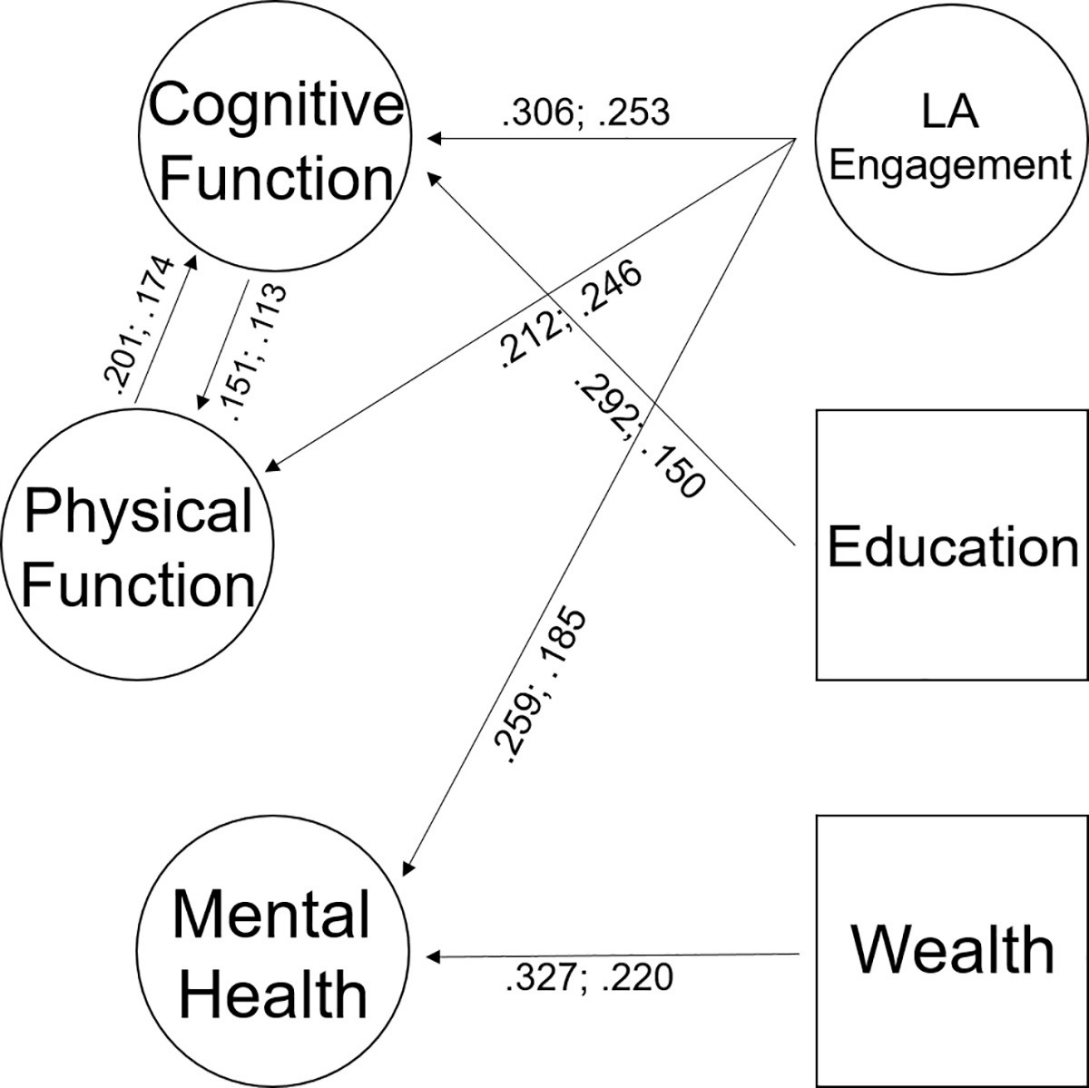
Representation of the significant paths in the SEM model. The numbers indicate the standardized path coefficients in males and females, respectively. Non-significant paths in either males or females and the indicators of the latent variables (all significant) are omitted for the sake of clarity.

The latent variables and regression coefficients are summarized in Table 2. LA engagement was positively related to all the latent factors in both male and female participants (all *p*s ≤ .003). The size of the effects was moderate (Std.Est ranging between 0.185 and 0.306; Table 2), yet comparable or even superior to the one of education and wealth. Also, the results showed a statistically significant reciprocal effect between cognitive function and physical function (Std.Est ranging between 0.113 and 0.201). No significant effect was observed between mental health and physical function or cognitive function. Finally, no meaningful difference was observed with the WLSM estimator (see the OSF link). Fig 2 highlights the statistically significant paths in the SEM model.

**Table 2:** The results of the SEM model.

	*Males*					*Females*				
Latent variables										
	*Estimate*	*Std*.*Err*	*p-value*	*Std*.*Est*		*Estimate*	*Std*.*Err*	*p-value*	*Std*.*Est*	
*Cognitive Function*										
MoCA-J	1.000			0.672		1.000			0.596	
ADAS Recall	0.854	0.104	.000	0.587		0.920	0.182	.000	0.552	
Number Series	0.590	0.081	.000	0.405		0.603	0.126	.000	0.353	
*Physical Function*										
Chair Stand Test	1.000			0.589		1.000			0.646	
Gait Speed	0.867	0.104	.000	0.498		0.836	0.122	.000	0.547	
Stepping Test	1.522	0.190	.000	0.854		1.082	0.164	.000	0.732	
*Mental Health*										
WHO5	1.000			0.727		1.000			0.696	
Positive Affect	1.103	0.115	.000	0.808		1.079	0.150	.000	0.764	
Life Satisfaction	0.997	0.102	.000	0.719		0.882	0.115	.000	0.617	
**Regressions**					*R*^*2*^					*R*^*2*^
*Cognitive Function*					.427					.204
LA Engagement	0.219	0.063	.000	0.306		0.163	0.054	.003	0.253	
Physical Function	0.228	0.047	.000	0.201		0.160	0.042	.000	0.174	
Mental Health	0.015	0.048	.745	0.017		-0.082	0.038	.034	-0.096	
Education	0.267	0.088	.002	0.292		0.131	0.081	.108	0.150	
Wealth	0.081	0.049	.096	0.103		0.041	0.064	.527	0.055	
*Physical Function*					.199					.132
LA Engagement	0.134	0.045	.003	0.212		0.172	0.046	.000	0.246	
Cognitive Function	0.133	0.036	.000	0.151		0.123	0.030	.000	0.113	
Mental Health	^a^-0.003	0.026	.896	-0.004		0.031	0.027	.251	0.034	
Education	0.030	0.062	.621	0.038		0.011	0.071	.878	0.011	
Wealth	0.093	0.049	.057	0.127		-0.003	0.055	.952	-0.004	
*Mental Health*					.214					.114
LA Engagement	0.203	0.053	.000	0.259		0.140	0.045	.002	0.185	
Cognitive Function	0.002	0.030	.945	0.002		-0.070	0.025	.005	-0.059	
Physical Function	^a^-0.003	0.026	.896	-0.004		0.033	0.024	.165	0.031	
Education	0.008	0.075	.915	0.008		0.008	0.072	.912	0.008	
Wealth	0.297	0.058	.000	0.327		0.192	0.057	.000	0.220	

*Note*. Estimate = unstandardized path coefficient; Std.Err = standard error; *p*-value = significance level; Std.Est = standardized path coefficient.

^a^ An equality constraint was applied to identify the model.

## Discussion

This paper quantified the impact of LA engagement on measures of successful aging including cognitive function, physical function, and mental health in a sample of Japanese older adults. LA engagement appears to be positively related to all these three constructs in both men and women (all *p*s ≤ .003). The implementation of a comprehensive SEM model allowing to consider different successful aging indicators concurrently, the excellent goodness of fit, the high statistical power, and the implementation of latent constructs based on tasks measuring objective performance (cognitive function and physical function) and reliable self-reported questionnaires (mental health) make the findings of the present study more reliable than most of previous research.

### Substantive findings

Overall, the present results corroborate the idea that LA engagement contributes to explaining the individual differences in cognitive function, physical function, and mental health. LA engagement was positively associated with successful aging indicators. The results are in line with the “use it or lose it” theoretical framework and previous findings in Western populations [3,13,48]. Thus, the findings of the present study support the position that leading an active lifestyle, here assessed by engagement in leisure activities, is a universal and culture-independent means contributing to successful aging, invariant across different countries and cultures.

Inspection of the regression standardized path coefficients (Std.Est column in the Table 2) indicates that the impact of LA engagement on the three latent variables is statistically significant and comparable (or even superior; range of Std.Est 0.185–0.306) to the one exerted by education and self-perceived wealth. Furthermore, the impact of LA engagement is systematically superior to the one exerted by the three endogenous variables on each other (range -0.096–0.201). Nonetheless, the size of these effects is somewhat smaller than the ones reported in those few studies implementing an SEM approach (e.g., 0.490; [3]). This discrepancy is probably due to the inclusion of three endogenous latent variables connected to each other with feedback loops in a single non-recursive model. Such a model controls for the potential confounding effects of one latent variable on the others. Part of the variance that it would be intercepted by LA engagement is instead absorbed by the paths connecting the three latent variables. Simply put, the model controls, for example, that the positive relationship between LA engagement and cognitive function does not only stem from better mental health or physical function. That being said, overall, LA engagement appears to contribute significantly to explaining unique variance in individual differences in important aspects of successful aging.

Some indirect effects were observed for LA engagement, specifically when considering cognitive and physical function. In particular, cognitive function and physical function influenced each other in a feedback loop, which supports recent evidence showing that the relationship between cognition and physical health is bidirectional [29,30]. LA engagement thus seems to exert an indirect effect on cognitive function mediated by physical function and vice versa. On this regard, the benefits of physical activity on cognitive function can be attributed to an ameliorated overall health condition (e.g., brain oxygenation and stimulation of neurogenesis [49]). Why better cognition should lead to improved physical health is less obvious. A possible explanatory mechanism could be an overall better self-regulation enabling the participant to be more physically active [29]. However, due to the correlational nature of the present study, no causal inference in either direction can be made and, thus, none of these hypotheses can be tested.

By contrast, mental health was not associated with either of the other two latent variables. In particular, the absence of any link between physical function and mental health appears to contradict previous findings in the field [17,50]. Possibly, this discrepancy is explained by the use of subjective measures of physical function in prior studies. In fact, while self-reported measures of physical function and mental health may reflect individuals’ perception of their overall health condition, objective measures of physical function may not be correlated with subjective measures of mental health. It is further of note that we used exclusively balance and gait measures as indicators of physical function, which may also have contributed to the lack of relation between our physical and mental health latent construct. That being said, the topic is certainly worth of further investigation.

Finally, there is no clear evidence that the effect of LA engagement on the three latent constructs differed between males and females. The relevant standardized path coefficients were relatively homogeneous when comparing the patterns across genders. A notable difference between the models for males and females was, however, the amount of explained variance, which was nearly twice as large in males compared to females. This is probably because the covariates were associated with greater factor loadings in males than in females. Basically, considering gender through multi-group testing improved the goodness of fit but had no notable impact on the relationship between LA engagement and the endogenous variables (i.e., cognitive function, physical function, and mental health).

### Recommendations for future research

In this study, we have considered LA engagement as a single trait. This assumption is upheld by the high internal consistency of our LA questionnaire (Cronbach’s *α* = .88) and is in line with substantial findings in the field [3]. Nonetheless, it is possible that engagement in particular types of activities (e.g., playing strategy games, technology use) is more strongly linked to specific constructs such as cognitive function [10,11,33,48]. In future studies, it is thus recommendable to test whether overall LA engagement subsumes specific types of activities impacting differently on the examined constructs, or whether specific activities are more important for certain successful aging aspects than others. The main drawback of this approach is that requires larger sample sizes than the ones usually included in surveys. Reducing the number of activities in the questionnaire is an alternative [2] but it has the serious shortcoming of decreasing the overall reliability of the measure. In fact, some individuals may practice relatively unusual LAs that would be necessarily excluded in a shorter questionnaire.

Another point of interest is the frequency of LA engagement. The sheer number of activities practiced by older adults is certainly a good proxy for their engagement in LA, but it is not necessarily the best possible one. How often an activity is practiced may play a significant role too. A further improvement may thus be, for instance, the use of a Likert scale to assess the frequency with which the participants engage in the activities they practice [3,33]. It is worth noting, however, that including frequency may not meaningfully enhance the predictive power of the measure. In fact, previous research suggests that most health benefits of an active life-style in the elderly occur after only a moderate amount of engagement [51].

Finally, a fundamental caveat needs to be mentioned. Our analysis is correlational in nature, and thus cannot establish any direction of causality between LA engagement and measures of successful aging. In our opinion, there are three possible explanations for our results: (a) LA engagement *causes* improvements in cognitive function, physical function, and mental health; (b) people with superior cognitive function, physical function, and mental health, are more likely to engage in LA; and (c) LA and the three constructs influence each other, that is, LA engagement positively affects measures of successful aging which, in turn, promotes LA engagement. This latter possibility is, in our opinion, the most likely explanation. That being said, studies implementing an LA intervention are necessary to test this hypothesis. Specifically, a longitudinal non-recursive SEM model would allow researchers to assess this presumed “virtuous circle” between LA engagement and measures of successful aging by imposing a feedback loop between the variables at different time points.

### Conclusions

The present study reported an SEM model examining the relationship between LA engagement and three essential dimensions of successful aging (i.e., cognitive function, physical function, and mental health) in a large sample of Japanese older adults. In line with substantial research into the field, the results confirmed the link between LA engagement and cognitive function. However, the size of the effect was meaningfully smaller than the one reported in previous studies. Similar effects were found for physical function and mental health.

The investigation significantly extends our knowledge in the field. First, thanks to a more comprehensive modeling approach, the study provides a more reliable estimate of the impact of LA engagement on cognitive function. Second, due to the use of multiple and objective physical indicators, it adds much-needed evidence of a link between LA engagement and preservation of good physical function in the elderly. Similar considerations apply to the influence of LA engagement in older adults’ mental health. Third, the findings suggest that the role played by LA engagement is cultural-independent. Finally, our study sheds some light on mechanisms of LA engagement that have not been much (if not at all) investigated so far, such as the bidirectional effects of practicing LAs on cognitive function and physical function.
